# vigiRank for statistical signal detection in pharmacovigilance: First results from prospective real‐world use

**DOI:** 10.1002/pds.4247

**Published:** 2017-06-27

**Authors:** Ola Caster, Lovisa Sandberg, Tomas Bergvall, Sarah Watson, G. Niklas Norén

**Affiliations:** ^1^ Uppsala Monitoring Centre WHO Collaborating Centre for International Drug Monitoring Uppsala Sweden; ^2^ Department of Computer and Systems Sciences Stockholm University Kista Sweden

**Keywords:** logistic regression, postmarketing surveillance, predictive modelling, spontaneous reports, strength of evidence

## Abstract

**Purpose:**

vigiRank is a data‐driven predictive model for emerging safety signals. In addition to disproportionate reporting patterns, it also accounts for the completeness, recency, and geographic spread of individual case reporting, as well as the availability of case narratives. Previous retrospective analysis suggested that vigiRank performed better than disproportionality analysis alone. The purpose of the present analysis was to evaluate its prospective performance.

**Methods:**

The evaluation of vigiRank was based on real‐world signal detection in VigiBase. In May 2014, vigiRank scores were computed for pairs of new drugs and WHO Adverse Reaction Terminology critical terms with at most 30 reports from at least 2 countries. Initial manual assessments were performed in order of descending score, selecting a subset of drug‐adverse drug reaction pairs for in‐depth expert assessment. The primary performance metric was the proportion of initial assessments that were decided signals during in‐depth assessment. As comparator, the historical performance for disproportionality‐ guided signal detection in VigiBase was computed from a corresponding cohort of drug‐adverse drug reaction pairs assessed between 2009 and 2013. During this period, the requirement for initial manual assessment was a positive lower endpoint of the 95% credibility interval of the Information Component measure of disproportionality, observed for the first time.

**Results:**

194 initial assessments suggested by vigiRank's ordering eventually resulted in 6 (3.1%) signals. Disproportionality analysis yielded 19 signals from 1592 initial assessments (1.2%; *P* < .05).

**Conclusions:**

Combining multiple strength‐of‐evidence aspects as in vigiRank significantly outperformed disproportionality analysis alone in real‐world pharmacovigilance signal detection, for VigiBase.

## 1 INTRODUCTION

Individual case reports of suspected harm from medicines remain the primary source to uncover risk with medicines after they have been approved for broader use.^1^ For many national and international organisations, statistical methods have become crucial to help prioritise the clinical assessment of potential safety signals. In practice, disproportionality analysis^2^ is the state‐of‐the‐art approach to statistical signal detection for pharmacovigilance, despite being based entirely on statistical associations, disregarding the strength of individual reports. This is clearly contrasted by manual clinical assessment, which carefully considers report quality^3^ and attempts to account for all relevant aspects of the reported information.^4^

**Figure 1:**
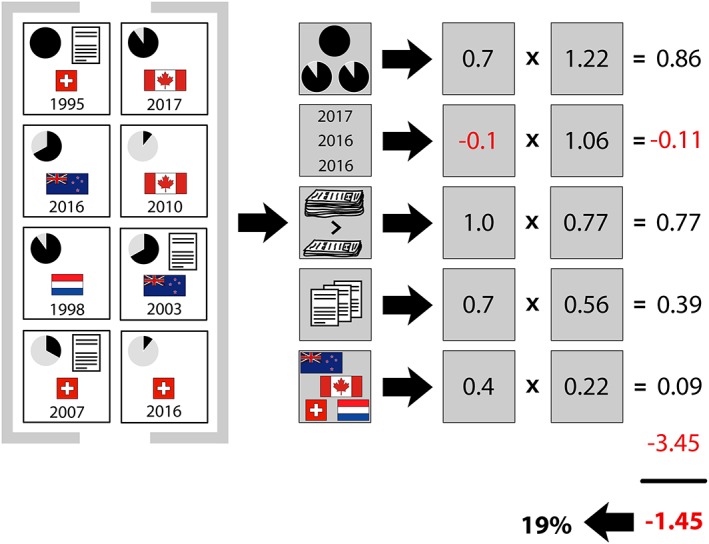
vigiRank computes a score for each considered drug‐ADR pair and then ranks all pairs based on their respective score from highest to lowest priority in the subsequent clinical assessment. In this fictional example, there are 8 reports on a given drug‐ADR pair, conceptually summarised in the leftmost section. For example, the top left report is Swiss, includes a case narrative, attains a vigiGrade completeness score^6^ of 1.0, and was received in 1995. The second section shows the raw data for each of the predictors: 3 informative reports (completeness score ≥ 0.9), 3 recent reports (entered during the last 3 years), disproportionality = TRUE (standard global IC analysis^7^, ^8^ or a locally disproportional pattern^9^), 3 reports with case narratives, and 4 countries of origin with positive IC.^7^, ^8^ The third section displays how the raw data are transformed and multiplied with their corresponding data‐driven coefficients. The fourth section sums the independent contributions from all variables with the intercept (−3.45) to produce the overall value of −1.45 on logit scale, corresponding to a probability of 0.19, denoted the vigiRank score. The logit value and the score are equivalent for ranking purposes. The theoretical maximum and minimum of the vigiRank score are 0.34 and 0.011, respectively. If only disproportionality is present, the resulting score is 0.023. Note that 2017 is used as the reference point to determine whether a report is recent. This figure has been reproduced from Caster et al^5^ with slight modifications. [Color figure can be viewed at wileyonlinelibrary.com]

In the hope of improving statistical signal detection performance, we recently devised a fundamentally different approach called vigiRank.^5^ vigiRank is a data‐driven predictive model for emerging safety signals that accounts for the completeness and recency of individual reports and their geographic diversity, alongside disproportional reporting and presence of narratives^5^ (see Figure 1). Other approaches that combine disproportionality with orthogonal information in a similar manner have followed.^10^

Retrospective evaluation against a set of historical safety signals from the European Medicines Agency indicated substantial improvement in signal detection performance with vigiRank compared to disproportionality analysis alone.^5^ Such evaluation is more relevant than the standard practice of evaluating performance with labelled adverse drug reactions (ADRs) as positive controls.^11^ However, until now, vigiRank's impact on prospective real‐world pharmacovigilance has not been known.

In 2014, vigiRank was adopted as the core statistical signal detection method for the Uppsala Monitoring Centre's (UMC's) analysis of VigiBase, and we are now in a position to evaluate its performance in guiding prospective signal detection compared to that observed historically for disproportionality analysis.

**KEY POINTS**

- vigiRank is a recently published novel approach to statistical signal detection that accounts not only for disproportionate reporting patterns but also for the completeness, recency, and geographic spread of individual case reporting, as well as the availability of case narratives.
- In this first prospective evaluation of vigiRank, its performance in global individual case reports was over 2.5‐fold better than disproportionality analysis alone, in terms of the proportion of initial assessments triggered by statistical signal detection that eventually resulted in signals.

## 2 METHODS

UMC signal detection is performed on behalf of the WHO Programme for International Drug Monitoring. The data are taken from VigiBase, the WHO global database of individual case safety reports.^12^

Since 2014, this process currently consists of 3 steps that are reiterated roughly every 6 months. In the first step, a scope is selected that determines a base list of drug‐ADR pairs to consider. For drug‐ADR pairs on this list, vigiRank scores are computed (see Figure 1) and other relevant information is extracted. In the second step, spanning a period of about 2 weeks, UMC research staff performs initial assessments of the drug‐ADR pairs on the list, working from the highest vigiRank score and downwards. In the initial assessment, each assessable pair is classified as either labelled, non‐signal, or worthy of in‐depth assessment. Any decision to move ahead to in‐depth assessment is verified by a medical doctor. In the third step, in‐depth assessment by internal or external clinical experts classifies pairs as signals or non‐signals. Both initial and in‐depth assessment also permit decisions to keep drug‐ADR pairs under review, awaiting further reporting. Here, a “signal” implies a definitive decision to disseminate the finding within the WHO Programme for International Drug Monitoring as a formal written communication. In addition, most signals are later made publicly available via the WHO Pharmaceuticals Newsletter.

All drug‐ADR pairs evaluated in this paper were reported at most 30 times and from at least 2 countries, with a restriction to new drugs (at most 5 years since first reported in VigiBase) and WHO Adverse Reaction Terminology critical terms. All in‐depth assessments were completed within 15 months of the initial data screen, which took place in May 2014.

The primary performance metric was defined as the proportion of drug‐ADR pairs subjected to initial assessment that eventually resulted in a signal. The secondary performance metric was the proportion of initial assessments that were deemed interesting enough to warrant in‐depth assessment.

The outcomes observed for vigiRank were compared to corresponding historical metrics from 2009 to 2013, when first‐pass screening of VigiBase relied on disproportionality analysis applied in quarterly database screens. One filter used during this period closely resembles the set‐up used for vigiRank and so was used as comparator. This filter identified pairs of new drugs and WHO Adverse Reaction Terminology critical terms that (1) were reported at most 30 times from at least 2 countries and (2) attained, for the first time, a positive lower 95% credibility interval endpoint of the Information Component (IC_025_).^7^, ^8^ Historical data were retrieved from an internal signal detection tracking database. Apart from the differences in statistical screening methodology, the signal detection process used during the 2009 to 2013 control period was relatively similar to the current one, with the exception of having a smaller and more homogeneous group of staff of healthcare professionals performing initial assessments over more extended periods.

## 3 RESULTS

**Figure 2:**
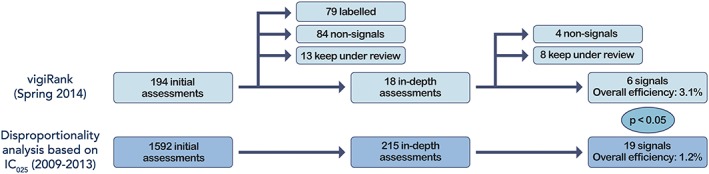
Prospective results from real‐world signal detection in VigiBase using vigiRank (upper section), compared to performance observed historically with disproportionality analysis (lower section). The reported *P* value was obtained using Fisher's exact test. Note that, unfortunately, information on labelledness and decisions to keep under review could not be reliably retrieved for the historical comparison period. IC, Information Component. [Color figure can be viewed at wileyonlinelibrary.com]

All results are presented in Figure 2. Overall, 194 drug‐ADR pairs (on 62 unique drugs and 96 unique ADRs) highlighted by vigiRank were subjected to initial assessment, resulting in 6 signals (3.1%) following the in‐depth assessments. These pairs covered the range of vigiRank scores from 0.34 (equal to theoretical maximum) to 0.061. The observed performance for vigiRank is over 2.5‐fold better than that seen historically for disproportionality‐ based signal detection, with 19 signals out of 1592 initial assessments on 287 unique drugs and 332 unique ADRs (1.2%; *P* < .05 using Fisher's exact test). The 6 vigiRank signals came out of 18 in‐depth assessments, corresponding to 9.3% of the initial assessments. The corresponding proportion for disproportionality analysis was 215 of 1592 (14%; *P* = .17).

## 4 DISCUSSION

This study provides empirical support that vigiRank, a recently devised method that simultaneously accounts for multiple strength‐of‐evidence aspects, offers higher real‐world signal detection performance than disproportionality analysis alone. This corroborates our earlier retrospective evaluation^5^ and lends support to the UMC's shift towards using vigiRank rather than disproportionality analysis as the basis for routine signal detection.

The main strengths of our study are its prospective nature for the vigiRank cohort, its overall size, and its independence of the data used for the development of vigiRank. Its main limitation is that there are several other factors varying between the two study periods beyond the choice between disproportionality analysis and vigiRank. Alongside the introduction of vigiRank in 2014 came a new operational framework for initial signal assessments at the UMC, which involves a larger and more heterogeneous group of staff for shorter and more intense periods. In addition, there has been staff turnover during the 5‐year period, including the in‐house medical doctors ultimately responsible for determining which combinations that merit in‐depth assessment. However, all of these factors should primarily affect decisions whether to perform in‐depth assessment and have little influence, if any, on signal classifications in those assessments. Since the observed difference in performance relates to the latter, these factors are unlikely explanations.

Although vigiRank offers great improvement overall compared to disproportionality analysis, it does not generate a higher rate of initial assessments being sent for in‐depth assessment by clinical experts. A possible explanation is that vigiRank proposes a set of drug‐ADR pairs for initial assessment that are of generally superior quality and so subconsciously raises the bar for what is going to be subjected to in‐depth assessment. The much more thorough and therefore labour‐intensive approach of the in‐depth assessments may be a contributing factor for such behaviour. Nevertheless, we are confident that it is the overall efficiency that is of real importance and the one that therefore should be measured.^13^

It is important to note that our comparison does not contrast the vigiRank score and IC measure in isolation but reflects specific applications for practical use. For the IC, we highlighted drug‐ADR pairs for which IC_025_ exceeded 0 for the first time. For vigiRank, we reviewed pairs in the order of descending scores. This could explain part of the substantial gain in performance, which exceeds that observed in the previous retrospective comparison.^5^ While the lowest observed vigiRank score among our assessed drug‐ADR pairs (0.061) may appear low, it is clearly higher than the vigiRank score corresponding to presence of only disproportionality (see Figure 1). Furthermore, in VigiBase globally, a vast majority of reported drug‐ADR pairs have lower vigiRank scores than this.

vigiRank is different from the majority of other efforts to advance statistical signal detection in pharmacovigilance. Most developments seem to have been made in the area of multivariate methods aiming to account for possible confounders, such as age or indication for use, to adjust and generalise the crude reporting associations offered by disproportionality analysis.^14^, ^15^ As noted elsewhere, this is orthogonal to the principles of vigiRank, and possible synergies might therefore be possible.^5^

A challenge with a more complex method like vigiRank compared to basic disproportionality analysis is that it becomes more difficult to apply in other data sets. Adaptation could be made at different levels, where refitting the underlying predictive model in the target database is the most ambitious and most likely to succeed.^5^ Also, whereas the vigiRank score itself may be opaque and not meaningful in clinical assessment, its individual components might. Specifically, disproportionality and geographic spread are aspects of strength of evidence in their own right.

In conclusion, combining multiple strength‐of‐evidence aspects as in vigiRank significantly outperforms disproportionality analysis alone in real‐world pharmacovigilance signal detection, for VigiBase. This is a first success story in need of independent verification, but the substantial improvement observed warrants careful consideration by anyone seeking to improve their statistical signal detection for individual case reports.

## CONFLICT OF INTEREST

The authors declare no conflict of interest.
